# Biomineral-Based Composite Materials in Regenerative Medicine

**DOI:** 10.3390/ijms25116147

**Published:** 2024-06-02

**Authors:** Sung Ho Kim, Mi-Ran Ki, Youngji Han, Seung Pil Pack

**Affiliations:** 1 Department of Biotechnology and Bioinformatics, Korea University, 2511 Sejong-ro, Sejong 30019, Republic of Korea; sungho509@korea.ac.kr (S.H.K.); allheart@korea.ac.kr (M.-R.K.); 2 Institute of Industrial Technology, Korea University, 2511 Sejong-ro, Sejong 30019, Republic of Korea; 3 Biological Clock-Based Anti-Aging Convergence RLRC, Korea University, 2511 Sejong-ro, Sejong 30019, Republic of Korea; youngjihan@korea.ac.kr

**Keywords:** biomineral, biomineral composites, regenerative medicine, hard tissue engineering

## Abstract

Regenerative medicine aims to address substantial defects by amplifying the body’s natural regenerative abilities and preserving the health of tissues and organs. To achieve these goals, materials that can provide the spatial and biological support for cell proliferation and differentiation, as well as the micro-environment essential for the intended tissue, are needed. Scaffolds such as polymers and metallic materials provide three-dimensional structures for cells to attach to and grow in defects. These materials have limitations in terms of mechanical properties or biocompatibility. In contrast, biominerals are formed by living organisms through biomineralization, which also includes minerals created by replicating this process. Incorporating biominerals into conventional materials allows for enhanced strength, durability, and biocompatibility. Specifically, biominerals can improve the bond between the implant and tissue by mimicking the micro-environment. This enhances cell differentiation and tissue regeneration. Furthermore, biomineral composites have wound healing and antimicrobial properties, which can aid in wound repair. Additionally, biominerals can be engineered as drug carriers, which can efficiently deliver drugs to their intended targets, minimizing side effects and increasing therapeutic efficacy. This article examines the role of biominerals and their composite materials in regenerative medicine applications and discusses their properties, synthesis methods, and potential uses.

## 1. Introduction

Humans may experience tissue degeneration and damage from a variety of sources, including diseases, injuries, trauma, and aging, necessitating regeneration to restore tissue functions [1]. These challenges significantly impair the quality of life of affected individuals and also contribute to escalating healthcare costs society-wide [2]. Regenerative medicine is a multidisciplinary field that aims to restore tissue functions. Researchers in the fields of materials science and engineering are engaged in active collaboration to develop composite materials using both natural and synthetic polymers, as well as minerals and metallic materials for a diverse range of medical applications, including tissue regeneration, orthopedics, dental materials, wound healing, and scaffolding [3,4,5].

In general, human tissues, including skin and bones, are not composed of a single material but, rather, a hierarchical composite structure. Consequently, materials for regenerative medicine, when composed of a singular material type, present challenges in accurately emulating the micro-environment of both hard and soft tissues and organs [6]. Conversely, composite materials are meticulously engineered to interface with biological systems, thereby facilitating the regeneration or replacement of tissues, organs, or any specific bodily functions [7]. Such composite materials, pivotal in regenerative medicine, are ingeniously composed of a confluence of minerals, polymers, and metallic elements, each tailored for specific therapeutic objectives [8,9].

The use of composite materials has gained attention due to the fact that each component material has different physical and chemical properties that amplify the inherent advantages of each material while mitigating its disadvantages [10,11]. Composite materials must accurately mimic the morphological, biochemical, and mechanical features of the tissue micro-environment in order to effectively contribute to regenerative medicine. This replication is essential for enhancing cell adhesion, proliferation, and differentiation [12]. Specifically, composite materials used in regenerative medicine must possess attributes such as biocompatibility, bioactivity, appropriate surface characteristics (e.g., porosity or roughness), structural resemblance to the target tissue, and necessary mechanical properties [13]. The presence of biominerals can significantly benefit composite materials, as they serve as important building blocks. Unlike regular minerals, biominerals are produced by living organisms through a process called biomineralization. In a broader sense, biominerals also include minerals produced by mimicking the biomineralization process. Biominerals can provide both biological activity and structural stability, which are often challenging to achieve with a single material. These biominerals play a crucial role in providing a stable structure and an environment that promotes cell adhesion, interaction, proliferation, and differentiation, as supported via various sources [9,14,15].

The selection of materials for composite materials is crucial, as it depends on their suitability for specific purposes [16,17,18]. Mineral components are often chosen to enhance mechanical and biological activity in the fabrication of composites [19,20]. Biominerals play a vital role in the regeneration of skin, bones, and other tissues, participating in numerous essential biochemical functions [21,22,23]. Regulating the particle size and surface charge of biominerals can be beneficial in building structural composites. In addition, controllable degradation rates are particularly advantageous for composites that require long-term structural integrity or drug delivery systems that require controlled release mechanisms [24,25]. Consequently, biomineral-based composites have found extensive application in tissue regeneration, including dental materials, orthopedics, drug delivery, and wound healing in regenerative medicine [6]. Biominerals in composite materials play a key role in these applications. This review introduces biominerals and biomineral-based composites, provides a concise overview of their utility in regenerative medicine, and examines recent trends in their application.

## 2. Biominerals

### 2.1. Definition and Types of Biominerals

Biominerals encompass a diverse array of materials crucial for various functions in living organisms. These biominerals include carbonates, oxides, hydroxides, phosphates, sulfates, sulfides, silicates, and organic crystals, with over 60 different types identified in nature [26]. Biominerals are composite materials that often have superior properties compared to their abiotically formed counterparts. They serve purposes such as structural support, protection, magnetic orientation, mechanical strength, ion storage, and even optical sensing. Biominerals are often formed through the process of biomineralization, where living organisms incorporate elements from their environment to create these essential materials. The intricate structures and compositions of biominerals reflect both the functional requirements of the organisms and the availability of minerals in their habitats.

**Figure 1:**
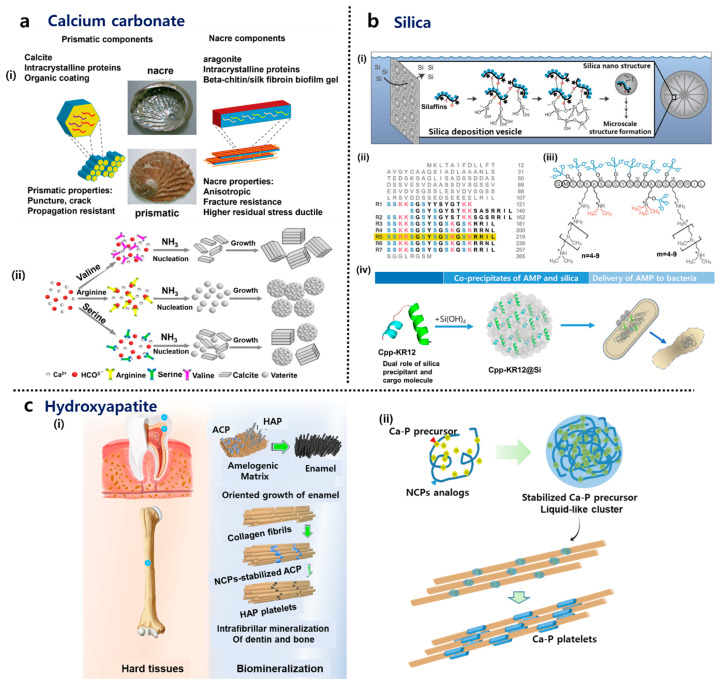
Biomineralization in living organisms and a scheme of biomineral-based materials that mimic the corresponding biomineralization mechanism. (**a**) (**i**). Construction and engineering scheme of mollusk shell layers. The prismatic (outer) and nacreous (inner) regions of the Pacific Red abalone (*Haliotis rufescens*) shell are presented for visual comparison. Reproduced from Ref. [31] with permission from Copyright © 2024 American Chemical Society. (**ii**). Possible formation mechanisms of the as-prepared CaCO_3_ in the presence of different amino acids. Reproduced from Refs. [32,33] with permission from Copyright © 2024, Springer Science Business Media B.V. (with permission from the Royal Society of Chemistry). (**b**) (**i**) Silica synthesis in diatoms. Post-translational modifications to silaffins include phosphorylation (blue circles), methylation (red lines), and polyamination (black stars). (**ii**) Primary amino acid sequence of the silaffin Sil1 protein. The mature part of the polypeptide is shown in bold, and the R5 peptide is highlighted. (**iii**) The R5 peptide from *C. fusiformis* with the native post-translational modifications. Reproduced from Ref. [34] with permission from © 2024 WILEY-VCH Verlag GmbH & Co. KGaA, Weinheim. (**iv**) Self-entrapment of antimicrobial peptide CPP-KR12 in silica matrix through CPP-KR12-mediated silica deposition [35] (CC By 4.0). (**c**) (**i**) The formation processes and mechanisms of human hard tissues, including bones and teeth [36] (CC By 4.0) with permission from Copyright © 2024, The Author(s). (**ii**) Biomineralization-inspired analogs of non-collagenous proteins (NCPs) to construct intrafibrillarly mineralized collagen.

Calcium carbonates are the most essential minerals in the construction of skeletal elements in various invertebrate groups, including echinoderms, mollusks, and arthropods [27]. In contrast, hydroxyapatite (HAP) crystals are commonly found as the building blocks in the bones and teeth of vertebrates [28,29]. Silica is widely found in protists, plants, and animals [30]. An understanding of the mechanisms through which organisms produce biominerals is of significant importance. Insights into the mechanisms of biomineralization can inform the development of biomaterials with enhanced mechanical performance and multifunctionality. In the following section, we will primarily focus on these three most common biogenic minerals (Figure 1).

### 2.2. Natural Occurrence and Formation of Biominerals

#### 2.2.1. Calcium Carbonate

Calcium carbonate (CaCO_3_) exists in various forms, with calcite, aragonite, and vaterite being the three primary polymorphs found in the skeletal elements of organisms. Calcite and aragonite are essential minerals in constructing skeletal elements in invertebrate groups like echinoderms, mollusks, and arthropods [27]. The abalone shell is composed of calcite (in the prismatic layer) and aragonite (in the nacreous layer). Vaterite, also known as μ-CaCO_3_, is less prevalent in nature due to its least thermodynamically stable polymorphic form. In an aqueous solution, vaterite can rapidly transform into calcite and aragonite [37]. Prior to calcite or aragonite, calcium carbonate formation involves the stabilization of the precursor phase, amorphous calcium carbonate (ACC), which is crucial for mineralization processes. Acidic polypeptides are essential in stabilizing ACC. In the presence of magnesium (Mg), proteins help in the stabilization of ACC, reducing the amount of acidic polypeptides required for ACC formation. Proteins extracted from sea urchin larval spicules have been utilized in in vitro experiments to replicate the biomineralization process, demonstrating the significance of these proteins in mineral formation [38]. Proteins extracted from sea urchin larval spicules play a vital role in biomineralization processes, particularly in the stabilization and transformation of ACC into crystalline calcium carbonate minerals. The transformation pathway of ACC to crystalline calcium carbonate minerals involves several steps, including the dehydration of hydrated ACC (ACCH_2_O) to form the stable crystalline phase, mainly calcite. Although organic materials are crucial in biomineralization processes, dissolved foreign inorganic ions, such as Mg^2+^ and (PO_4_)^3−^, commonly found in natural settings, can adsorb onto the surface or be incorporated into the structure of ACC. This, in turn, slows down the crystallization process [37].

Although aragonite structures with excellent mechanical performance are found in nature, such as nacre, experiments in synthetic systems have demonstrated that only a fraction of aragonite is produced compared to calcite under ambient conditions and in the absence of additives. The effectiveness of Mg^2+^ additives has been well established [39]. Soluble acidic macromolecules extracted from aragonite-forming tissues also contribute to aragonite formation [40]. While the detailed mechanisms remain unclear, they are generally attributed to interactions between acidic functional groups of biomacromolecules and mineral components. Zeng et al. demonstrated that aragonite formation is significantly promoted in a confined environment [41]. For instance, when the same concentrations of Mg^2+^ and SO_4_^2−^ were used as additives, the percentage of aragonite in the bulk solution was only 7%, but in 200 nm pores, this value increased to 69% and reached 100% in 50 nm pores. Even in the absence of additives, pure aragonite crystals were obtained when using smaller 25 nm pores. Zhang et al. found that nacre, typically considered a tough armor, has an anomaly in its energy dissipation ability [42]. It shows good energy dissipation only at low impact velocities, but at higher velocities, it has lower impact resistance compared to laminated structures. This anomaly depends on the structure’s size and boundary conditions. To address this, they proposed a hybrid architecture design strategy to optimize impact resistance at various velocities.

#### 2.2.2. Hydroxyapatite (HAP)

Bone is composed of HAP crystals and protein, primarily collagen. This combination of materials renders bone strong and flexible, enabling it to withstand impacts without breaking. Collagen forms long rods that create a robust network, while other non-collagenous proteins (NCPs) composed of approximately 180–200 different molecules contribute to the network’s strength and facilitate mineral attachment [43]. Negatively charged NCPs act as templates for mineral nucleation and growth, guiding the formation of hydroxyapatite (HAP) crystals within the collagen matrix [44]. By interacting with collagen and mineral phases, NCPs influence the spatial distribution and organization of mineral crystals within the collagen fibrils. The amorphous calcium phosphate (ACP) precursor, a transient phase during mineralization, is stabilized due to polyelectrolytes like negatively charged NCPs to facilitate intrafibrillar mineralization because the fluidic character of the amorphous precursor phase enables it to be drawn into the nanoscopic gaps and grooves of collagen fibrils via capillary action [45]. The amorphous-phase strategy offers advantages such as fluidity, efficient mineralization, and reorganization flexibility, making it crucial for material design and construction for bone repair. The spatial confinement effect of collagen fibrils on mineral growth leads to the nucleation of mineral crystals in gap zones within the fibrils, resulting in the formation of plate-shaped nanocrystals [46]. Mineralized collagen fibrils in bone play a crucial role in enhancing the material’s energy dissipation and fracture resistance characteristics beyond what the individual constituents can achieve. The mineral crystals within the collagen network bear a significant amount of stress, up to four times that of the collagen fibrils, while collagen primarily contributes to the material’s deformation response [47].

Dentin and enamel biomineralization processes involve the controlled formation of HAP crystals within a collagen matrix of these hard tissues. Dentin is tough like bone because it has a similar tiny structure with minerals organized inside collagen fibers. Odontoblasts play a crucial role in the production and secretion of unmineralized collagen, proteoglycans, and NCPs, which include dentin sialophosphoprotein and dentin matrix protein 1 (DMP1) [36,48]. NCPs, with their abundant carboxylic acid and phosphate functional groups, act as preferential sites for mineral nucleation and subsequent crystallization [49]. Even though NCPs make up a small portion of the organic matrix of dentin, their role in regulating and promoting the intrafibrillar mineralization of collagen fibrils is pivotal [50].

Matrix proteins, including amelogenin, ameloblastin, and enamelin, are of great importance in enamel development [51]. They regulate the biomineralization process and ensure the formation of a highly mineralized tissue. These proteins control the shape and arrangement of enamel crystals, with amelogenin being the most abundant enamel matrix protein [52]. Proteolytic products of amelogenin regulate apatite mineralization, forming nanoribbons that template the oriented growth of apatite fibers to form enamel rods during the maturation stage [53]. The cleavage and degradation of the proteinaceous matrix through proteolytic processes are essential in achieving enamel with a high degree of mineralization [54]. Matrix metalloprotease-20 (MMP-20) plays a critical role in enamel development by preventing the occlusion of undegraded amelogenin inside enamel crystals, ensuring the proper size, morphology, and crystallinity of enamel crystals [55].

The calcium phosphate polymer-induced liquid precursor (CaP-PILP) system mimics the intrafibrillar mineralization process in bone, replicating the natural mineralization mechanisms observed in biomineralization processes. This system utilizes bio-inspired artificial materials to stabilize intermediate mineral phases involved in biomineralization. This system employs poly-aspartic acid and polyacrylic acid to replicate the mechanisms of NCPs [56], while recent studies have also utilized synthetic DNA aptamers with specific secondary structures for collagen mineralization [57]. By increasing the concentration of mineral ions and polymeric additives, the CaP-PILP system achieves a viscous and transparent material with good fluidity, enabling it to penetrate and mineralize osteoporotic bone efficiently, resembling the natural mineralization processes in bone tissue.

#### 2.2.3. Silica

Diatoms are single-celled photosynthetic eukaryotes that are crucial to global primary production and carbon fixation, contributing over 20% of the Earth’s carbon fixation. They are abundant in marine and freshwater ecosystems, playing a vital role as primary producers in planktonic and benthic food webs [58]. Furthermore, the ability of diatoms to make silica-based cell walls called frustules has been a subject of fascination for centuries. Silica deposition in diatoms primarily occurs within a membrane-bound compartment known as the silica deposition vesicle (SDV). Silicic acid transport mechanisms are crucial for diatom silicification, as they determine the availability of silicic acid for the process. The intracellular stabilization of silicic acid at high concentrations is essential for maintaining the dynamic process of diatom cell wall formation [59]. Studies have shown intracellular silicic acid concentrations exceeding their saturation limit (greater than their 2 mM saturation limit), indicating the presence of unidentified organic compounds that prevent polymerization [60]. Silaffin acts as a template for silica deposition, binding to silicic acid and initiating polymerization [61]. The kinetics of silica polymerization are modulated via polyamines, which in turn influence the growth and patterning of silica structures in diatom cell walls [62]. Silacidine interacts with other biomolecules like silaffins and polyamines, influencing the kinetics of silica polymerization and the organization of silica structures in diatom cell walls [58]. Silicanin, which is the first identified SDV transmembrane protein, serves as a catalyst for the polymerization of silicic acid, facilitating the controlled deposition of silica and ensuring the precise development of the intricate cell wall structures in diatoms [63]. The collaboration of these biomolecules ensures the controlled and species-specific formation of the silica frustules in diatoms [58].

Silicatein-α, an abundant protein in the axial canal of sponges, plays a crucial role in silica deposition [64]. It is produced intracellularly in the sclerocytes, indicating its secretory nature. Mature silicatein-α undergoes post-translational modifications, including phosphorylation and dehydroxylation, before being transported into silicasomes to form siliceous spicules. The catalytic site of silicatein-α consists of a triad of serine, histidine, and asparagine residues [65]. A model suggests that histidine binds and stabilizes deprotonated silicic acid, initiating silicic acid polymerization in a solution. Silicatein-α’s catalytic action involves significant proton shuttling facilitated via water molecules, resulting in a negative charge on a neighboring oxygen atom, ultimately leading to the polymerization of silicic acid in sponges. Silintapin-1 is believed to not only boost the enzymatic activity of silicatein-α but also expedite the non-enzymatic polycondensation of the silica product before the complete synthesis of the biosilicon polymer is released [66].

Plants, including grasses, have the remarkable ability to mineralize silica in the apoplast, which may contain a super-saturation of 8 mM or more of silicic acid [67,68]. This process involves the secretion of mineralizing proteins into the silicic acid-rich environment, leading to the formation of silica outside the cell membrane. Plant cell wall polysaccharides [69], proteins [70], and lignin [71] are known to play a central role in cell wall local silicification. A key protein in this process is Siliplant1 (Slp1), which plays a crucial role in precipitating silica in sorghum silica cells [72]. After overexpression in *Escherichia coli*, purified Slp1 rapidly precipitates silica from a supersaturated silicic acid solution (approximately 91 mM). Notably, Slp1 does not exhibit sequence homology with biosilicification-related proteins in diatoms or sponges. Slp1 is a secretory protein that interacts closely with silica, decorating its surface and possibly being occluded within it [72]. In young leaves, silica cells initiate mineralization independently of local transpiration, resulting in porous and hydrated silica, which does not have any micrometric or sub-micrometric patterns, as seen in diatom frustules [73].

The field of biomimetic silica formation has been becoming increasingly intriguing due to its potential to utilize gentle reaction conditions, control the silica structure, and facilitate relatively simple cargo loading [74]. The advancement of bio-inspired and biomimetic silica formation has been fueled by the identification of the molecules involved in natural silica biomineralization processes like silaffin peptides and polyamines [35,75,76,77,78].

## 3. Classification of Biomaterials Used in Regenerative Medicine

### 3.1. Polymer Materials

A natural polymer, also termed a biopolymer, constitutes a polymer sourced from living entities, including plants, animals, and microorganisms [79]. Notable examples of natural polymers encompass carbohydrates—such as chitosan, alginic acid, starch, and cellulose—and proteins—such as gelatin, collagen, hyaluronic acid, and fibrin. Nanocellulose (NC) merges the significant characteristics of cellulose with the attributes of nanomaterials, thereby creating fresh opportunities for the field of materials science and its practical implementations [80]. The material possesses a large specific surface area, a high aspect ratio, and exceptional mechanical properties [80]. NC, with its unique structures, excellent properties, and abundant availability, has been extensively studied as a crucial component in the development of diverse materials including aerogels, sensors, pharmaceuticals, chiral materials, and catalysts [80]. Nanocellulose has been utilized in the production of medical devices, wound-healing materials, bioactive implants, and self-healing materials [81,82]. In contrast, synthetic polymers represent a distinct category of polymeric materials, predominantly comprising polyesters, and are extensively utilized in tissue engineering applications [83]. Synthetic polymers, including polylactic acid (PLA) [84], polyglycolic acid (PGA) [85], polycaprolactone (PCL) [86], and poly(lactic-co-glycolic acid) (PLGA) [87], are characterized by their biodegradability and biocompatibility. Moreover, they share several properties with petroleum-based plastics that render them suitable for industrial applications, such as mechanical strength, physical durability, biocompatibility, and processability [84,85,86,87]. Recent advancements in tissue engineering have concentrated on the development of scaffolds from polymer materials capable of emulating the extracellular matrix (ECM). These scaffolds aim to enhance cells’ adhesion to biomaterials and support new tissue formation during the process of tissue regeneration [88]. Polymers, owing to their inherent attributes like biodegradability and biocompatibility, present a myriad of advantages for the fabrication of regenerative products, thereby establishing themselves as materials of choice in this field [15].

Natural polymers exhibit inherent property variations stemming from their natural origins, which can result in challenges related to supply consistency and quality control. Additionally, they may possess processing limitations when compared to synthetic alternatives [89]. In contrast, synthetic polymers can pose environmental concerns due to their lack of biodegradability and the production of by-products. Additionally, if not appropriately engineered, they have the potential to cause negative effects in biomedical applications. The sustainability of certain synthetic polymers is a major concern due to their heavy reliance on petroleum-based resources [90]. In general, polymers have notable benefits in terms of their ability to work well with living organisms and their wide range of uses, especially in the area of creating artificial tissues. Nevertheless, there are still obstacles to overcome in order to guarantee the long-term viability of production, maintain a uniform level of quality, and ensure environmental safety.

### 3.2. Metallic Materials

Metallic biomaterials are extensively employed in the fields of dentistry and orthopedics, serving as substitutes for damaged or healing bone to support and enhance the healing process [91]. Commonly, standard surgical implants are fabricated from stainless steel, cobalt-based alloys, and titanium alloys. Nevertheless, these metallic biomaterials present several disadvantages, including the potential release of toxic metallic ions and/or particles as a result of corrosion and wear processes. Such releases may provoke inflammation and allergic reactions, thereby adversely affecting biocompatibility [92]. Furthermore, due to their mechanical properties, which differ significantly from those of natural bone tissue, these materials often provide inadequate stimulation for new bone growth. In contrast, titanium (Ti)-based metals, owing to their mechanical properties, which shape the memory effect, porous structure, and biocompatibility, have been identified as effective substitutes for bone [93]. High mechanical strength is an essential criterion for metallic biomaterials utilized in bone tissue engineering applications, where resistance to mechanical stress is imperative. Consequently, various fabrication methods have been developed to enhance biocompatibility and promote cell attachment [94].

### 3.3. Biomineral Materials

Various biominerals are fabricated by living organisms or synthetically, play important structural and functional roles, such as those of bones, teeth, and shells, and are used as regenerative medicine materials [19,95,96,97]. Though present in minor quantities within the body, these minerals are crucial for regenerating damaged tissue, maintaining skeletal integrity, and ensuring overall health. The source of these minerals is diverse, encompassing marine, soil, and animal origins, with their morphologies being influenced due to the specific methods of fabrication or purification employed. Minerals tailored for applications in tissue regeneration and drug delivery, whether derived artificially or naturally, are referred to as biominerals. The efficacy of biominerals is significantly impacted due to their biological properties, including bioactivity, biocompatibility, and resorbability. For an implant to be successful, the biominerals employed must exhibit biocompatibility and non-toxicity to the body’s surrounding cells [98]. While polymeric and metallic materials may provide the necessary spatial and mechanical support for cell attachment and proliferation, they typically lack biological activity and do not facilitate cell differentiation [99]. Conversely, biominerals can supply bioactive cations (e.g., Ca^2+^, Mg^2+^, and Si^4+^) and anions (e.g., OH^−^ and PO_4_^3^^−^), which are pivotal in the cell differentiation process within the body [100,101]. These bioactive ions serve as fundamental building blocks essential for the regeneration of bone and skin. Hydroxyapatite (HAP), mirroring the chemical and mineral composition of bone’s inorganic component, dominates as a primary constituent of bone tissue, as established through tissue engineering research. Calcium-based minerals, including calcium carbonate (CaCO_3_), β-tricalcium phosphate (β-TCP), and octacalcium phosphate (OCP), bear a resemblance to hydroxyapatite and play a vital role in providing a conducive micro-environment for bone regeneration [96,102]. The design of biomimetic scaffolds that emulate the mineralized structures of bones and teeth heavily relies on these biominerals. Furthermore, silicon is recognized as a vital trace element for human life, with its presence noted in the skeleton, blood vessels, heart, muscle, skin, hair, ligaments, cartilage, and internal organs such as the liver, lungs, and brain [103]. Silicon dioxide (SiO_2_), or silica, emerges as a critical material for scaffold and drug delivery systems. Its significance extends beyond being a silicon source; it also plays a role in inhibiting inflammatory responses, promoting cell differentiation, and facilitating the binding of bioactive molecules [104]. Hench introduced bioactive glass (BG) in 1969 as a material to help fix bones, made of 45% SiO_2_, 24.5% Na_2_O, 24.5% CaO, and 6% P_2_O_5_ [105]. BG helps bone healing by releasing helpful ions like Na, Ca, Si, and P and forming a layer that bonds well with bone, which is different from other bone repair materials. Since its discovery, BG has been used in many bone and soft tissue repairs, from experiments to real surgeries [106].

In contrast, biomineral materials, while promising, do have certain disadvantages. The morphologies of these minerals are influenced due to the specific methods of fabrication or purification employed, which could be complex and resource-intensive [107]. The source of these minerals is diverse, encompassing marine, soil, and animal origins, which could lead to variability in their properties [108]. There exists some controversy pertaining to the temperature effect on the biomineralization-forming process [109].

### 3.4. Composite Materials

Composite materials are commonly defined as those composed of two or more distinct substances, wherein there exists a significant disparity in either the chemical or physical properties between the components [110]. Composite biomaterials have enhanced the capabilities of existing materials by amalgamating the biological, chemical, and mechanical attributes of each constituent. Although single materials have been traditionally employed for specific purposes, the field of regenerative medicine has been profoundly influenced due to the advent of composite materials. The utilization of composite materials has led to substantial improvements in the recovery outcomes for patients with critical-size defects in their bones or skin, as well as in their quality of life and life expectancy [83,111]. Composites offer the flexibility to select materials tailored to specific applications, allowing for the development of constructs that closely emulate the requirements of particular tissues or organs [112].

**Figure 2:**
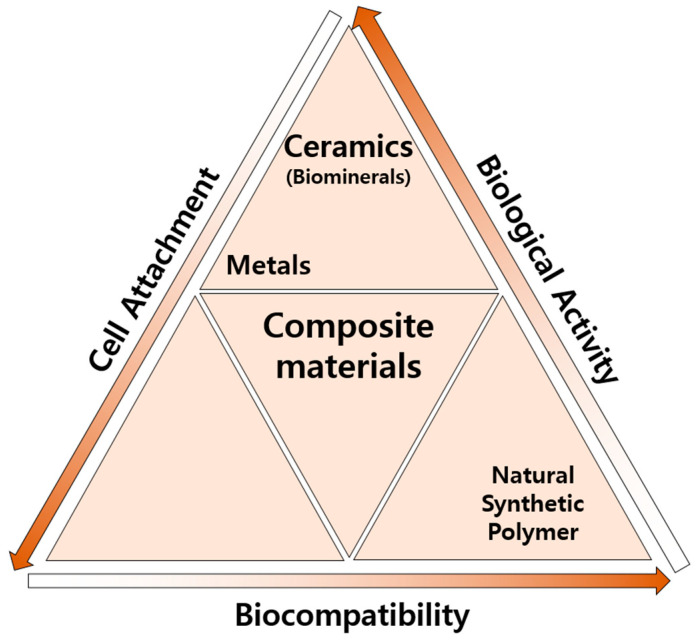
Characteristic triangular graph of biomaterials for regenerative medicine.

Constructed from biocompatible substances, composite materials interact favorably with biological systems (Figure 2), with each component compensating for the deficiencies of the others. This synergy enhances biocompatibility, potentially minimizing side effects and inflammation and facilitating the long-term integration of the composite material with the host tissue [113].

Moreover, based on their polymeric or biomineral constituents, composite materials can engage in electrostatic and Van der Waals interactions with bioactive molecules, such as growth factors and drugs, serving effectively as carriers for these substances [114,115]. The incorporation of bioactive molecules into composites allows for controlled release, thereby regulating immune responses, influencing cellular behavior, and promoting tissue regeneration. Spanning from tissue engineering to drug delivery, the composite materials utilized in regenerative medicine are distinguished due to their versatility, biomimetic capabilities, and mechanical properties. These attributes render them exceptionally suited to addressing the intricate challenges associated with tissue regeneration and repair [116].

Despite the numerous advantages of composites, it is important to consider the associated disadvantages [117]. The production of composite materials can be more intricate and expensive compared to that of single-material constructs. In addition, there is a potential for incompatibility between the constituents of the composite, which may impact the overall performance of the material. While composite materials are known for their strength, they can be more susceptible to impact damage than metals, which can compromise their structural integrity [118]. Furthermore, predicting the long-term behavior of composites, particularly in biological environments, can be challenging due to potential variations based on the interaction between their components.

### 3.5. Role of Biominerals in Enhancing the Properties of Composite Materials

**Figure 3:**
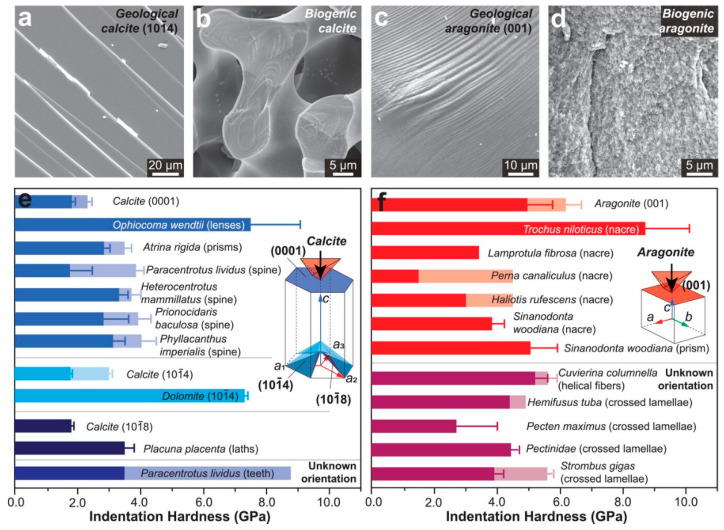
Mechanical difference between biogenic and geological minerals. (**a**,**b**) Scanning electron microscopy (SEM) images of the fracture surfaces of (**a**) geological calcite and (**b**) biogenic calcite (sea urchin spine, *Heterocentrotus mammillatus*). (**c**,**d**) Fracture surfaces of (**c**) geological aragonite and (**d**) biogenic aragonite (*Sinanodonta woodiana*). (**e**,**f**) Nanoindentation hardness of biogenic and geological (**e**) calcite and (**f**) aragonite along different crystallographic orientations. Reproduced from Ref. [27] with permission (CC BY 4.0). © 2024 The Authors. Advanced Science published by Wiley-VCH GmbH.

Biogenic minerals often have unique intracrystalline structural features like intracrystalline organic materials, trace element substitutions, and crystalline defects that enhance their mechanical properties, leading to higher hardness [27] (Figure 3). These features, including organic inclusions and residual stress/strain, play a significant role in strengthening biogenic minerals. Additionally, the presence of amorphous phases and nanograins in biogenic calcite and aragonite can also impact their hardness by impeding dislocations and improving the overall mechanical response. Furthermore, the smaller building blocks and aggregation of nanograins in biogenic aragonite create strong boundary effects, further increasing their hardness compared to geologic aragonite. By mimicking the features of biominerals, such as the nanostructuring of minerals and mixing with organics or other minerals, researchers reinforce the structure and improve the overall mechanical properties [119,120,121,122]. Specifically, inspired by the stabilized ACC of crustacean cuticles, Wu et al. reported the efficient ACC stabilization ability of the most abundant biopolymer cellulose nanofibers (CNFs) [123]. Through the cooperative stabilizing effect of a tight separation network between ACC and CNF surface carboxyl groups, CNFs exhibit long-term (>1-month) stability. In addition, The CNF/ACC composite films exhibited exceptional mechanical properties with a high strength of 286 MPa and toughness of up to 28.5 MJ/m^3^, surpassing synthetic biopolymer–calcium carbonate/phosphate composites.

Bone serves as a biomimetic model due to its lightweight structure and exceptional mechanical properties, particularly its remarkable fracture toughness that surpasses that of its basic constituents. The porous structures of natural materials, including traecular bone, sponge, and diatom skeletons, with their interconnected networks and hierarchical architectures, play a crucial role in distributing and dissipating applied stresses, thereby enhancing their overall mechanical properties and performance despite their light weight [124,125]. The interconnected pore network within the bone structure provides pathways for nutrient and waste transport, and it also allows for bone remodeling. By incorporating bio-inspired porous architectures into composite materials, it is possible to achieve a desirable combination of low weight and high mechanical strength [126]. Additive manufacturing, such as 3D printing, can be utilized to create intricate, hierarchical, porous structures inspired by natural materials [127,128,129]. Additionally, by combining materials like ceramics, polymers, and metals with bio-inspired, porous structures, we can enhance the strength-to-weight ratio of the final composite [130].

Balancing biodegradability with mechanical strength is crucial to ensure that biodegradable composites are environmentally friendly while still meeting performance requirements [131]. Metallic implants, while strong and ductile, can release toxic metallic ions into the body, necessitating the application of coatings to prevent this [132]. Calcium phosphate-based coatings are extensively researched due to their similarity to bone structure, promoting fixation and osseointegration [133,134]. The addition of bioactive minerals like Mg, Zn, and Sr into calcium phosphate phases enhances biocompatibility and accelerates biodegradability [135]. Biomineral-inspired colloidal liquid crystals are self-assembled materials created by combining organic polymers and inorganic crystals like CaCO_3_ or HAP, mimicking the hierarchical structures found in natural biominerals [136]. Due to their ability to self-assemble under mild and aqueous conditions, these materials offer environmentally friendly and biocompatible components, making them suitable for various applications in biomedicine and biotechnology [136]. Meanwhile, biominerals, such as titanium-doped HAP (Ti-HAP) offer a safer alternative for human and ecosystem health in sunscreen formulations [137]. Ti-HAP was nucleated on a gelatin/alginate matrix via nature-inspired biomineralization, yielding hybrid micrometric structures. Ti-HAP composites have high UV reflectance, low photoactivity, good biocompatibility, and an aggregate morphology that prevents dermal penetration. These materials are safe for topical application, eco-friendly, protect organic sunscreen components from degradation, and offer long-lasting UV protection.

## 4. Biominerals and Composite Materials in Regenerative Medicine

**Figure 4:**
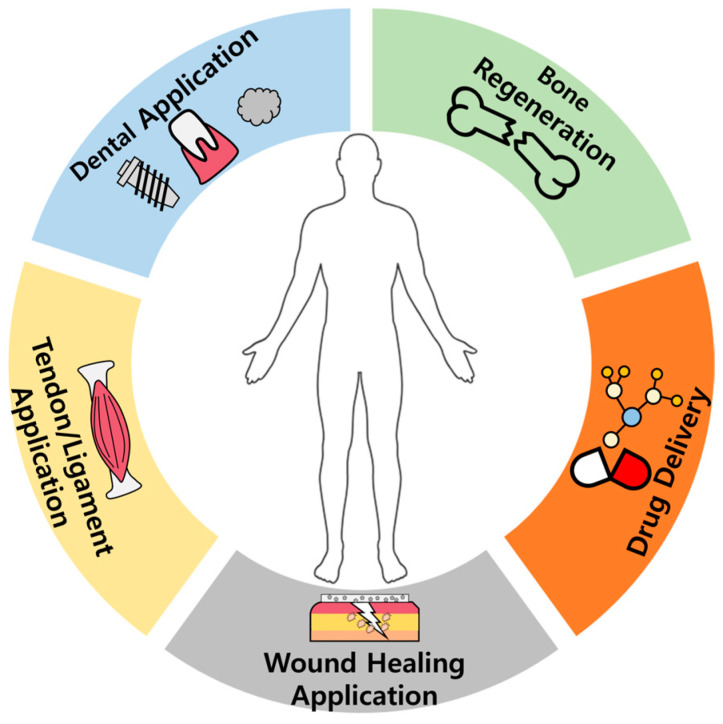
Application field of biominerals and their composite materials in regenerative medicine.

Biomineral-based composites have emerged as sophisticated solutions for applications across orthopedics, cartilage and bone prostheses in bone regeneration, tendons/ligaments, wound healing, and dental applications (Figure 4).

### 4.1. Bone Regeneration

In the field of tissue engineering, research is primarily focused on three key areas: seed cells, growth factors, and scaffold materials. Among these, scaffold materials are considered to be of paramount importance, as they not only provide a substrate for growth factors to adhere to but also promote cell proliferation [92]. From this perspective, scaffolds, by offering spaces conducive to cell growth, emerge as an effective therapeutic strategy for addressing critical-size defects and bone diseases [138]. Scaffolds tailored to bone defects are designed to facilitate cell growth while ensuring biocompatibility and biodegradability. The use of complementary composites in bone defects enhances bone formation and healing, with their synergistic interactions with other materials rendering them osteoconductive and osteoinductive [139].

**Figure 5:**
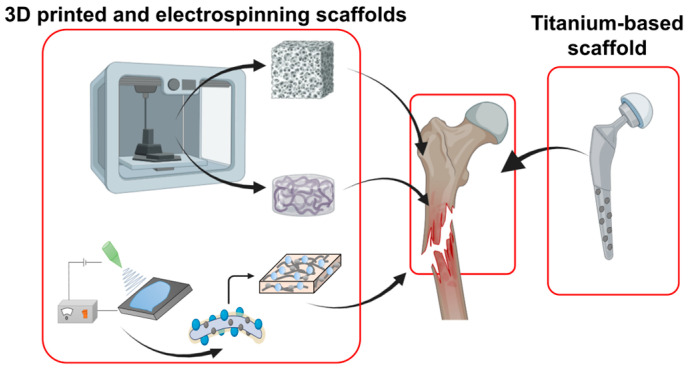
Application of biominerals in bone regeneration field using functionalized structure.

Historically, metallic materials have been used for internal fixation due to their durability and favorable physical properties [140]. Titanium-based metallic scaffolds, known for their robust physical properties, are commonly utilized for the hip bone [141]. However, they have disadvantages such as corrosion, stiffness, bioactivity issues, and the necessity for removal post-recovery [142]. In contrast, biominerals have been demonstrated to offset the disadvantages of metallic materials, enhancing cell adhesion and bioactivity [143]. Figure 5 depicts a methodology for the incorporation of biominerals to overcome the limitations of metallic materials. Metal-based biomineral composite scaffolds can be engineered into non-corrosive devices possessing the requisite strength to support damaged bone while also facilitating a strong bond formation between the bone and the scaffold [144]. Despite the biocompatibility of pure titanium, it requires a calcium-based mineral surface coating, such as HAP, for physiological activity and cell adhesion [145,146].

The advent of 3D-printed scaffolds marks a significant innovation, with their porous design enhancing oxygen and nutrient exchange and facilitating tissue regeneration. However, scaffolds crafted solely from natural polymers or bio-inks may suffer from inadequate mechanical and structural stability, often degrading too swiftly or unpredictably, impeding mass transfer and leading to tissue necrosis [147]. Conversely, highly durable scaffolds made from synthetic polymers or metals might degrade too slowly, potentially leading to the formation of a fibrous capsule and the impaired regeneration of the surrounding tissue due to a lack of integration of the scaffold with the host tissue [148]. A 3D-printed scaffold can be incorporated with bioceramics, or BGs, to create composite materials that mitigate the limitations of polymer scaffolds [106,149]. Biodegradable synthetic polymers like PCL, PLA, PLGA, and polyurethane (PU), as well as natural polymers, are commonly used. BGs offer several advantages, including stimulating angiogenesis, osseointegration, bone conduction, and compatibility [150]. Integrating biominerals such as BG into polymer scaffolds can allow for scaffolds with a proper physical micro-environment for the regeneration of bone [151].

Electrospun 3D scaffolds, composed of natural polymers, offer a structure suitable for the damaged area with excellent cell adhesion capabilities. Nevertheless, their low mechanical property and uncontrollable degradation rates render them unsuitable for hard tissue regeneration. The Pack group has developed a scaffold fabrication system that employs electrospun gelatin nanofibers with chimeric proteins capable of forming biominerals both calcium carbonate and biosilica for bone regeneration [152]. The integration of biominerals formed from multifunctional chimeric proteins into a polymer scaffold resulted in enhanced mechanical properties and bioactivity conducive to cell differentiation.

Polymer-based and biomineral-fabricated bone cement find applications in orthopedics, ranging from fracture treatment to artificial joint fixation [153]. The incorporation of nano-HAPs into natural polymeric composite scaffolds results in the optimal adhesion and bioactivity of cells, as well as enhanced mechanical properties [154]. Moreover, the incorporation of HAPs into biodegradable synthetic polymers enhances their biological activity, biocompatibility, and osteoconductivity [155,156]. These composites amalgamate the advantageous characteristics of their constituent materials while mitigating their individual drawbacks. Simulated body fluid (SBF) is employed to create 3D scaffolds designed with biominerals either coated on the surface or mixed as micro/nanoparticles [157]. Consequently, 3D composite scaffolds incorporating calcium- and silicon-based biominerals have demonstrated efficacy in hard tissue regeneration [36,158,159]. The versatility and design flexibility of biomineral composite scaffolds hold promise for various research and development endeavors in orthopedics and tissue regeneration.

### 4.2. Dental Applications

Oral health is defined as the absence of diseases and disorders that limit the ability to bite, chew, smile, and speak, as well as one’s psychosocial status [160]. Disease, injury, and congenital anomalies can adversely affect the dental system and, consequently, significantly reduce one’s quality of life [161].

Dental caries are a significant and irreversible dental disease. Therefore, it is important to halt the development of enamel caries’ early stages. By comparison, remineralization treatments using functional materials can inhibit and reverse enamel demineralization at an early stage, thereby restoring the damaged enamel [162]. Functional materials, including calcium phosphate, fluorinated compounds, and magnesium-related materials, can facilitate the deposition of calcium and phosphate ions or modify the solubility of HAP. Amorphous calcium phosphate (ACP) has been utilized in bio-inspired mineralization to repair tooth enamel. However, there are two main challenges in replicating the structure and strength of natural enamel using ACP. The first challenge is that the foreign ACP phase cannot initiate the epitaxial growth of enamel due to oversized ACP nanoparticles [163]. The second challenge arises from the use of polymeric additives to stabilize ACP, which can weaken the mechanical strength of the repaired enamel [36]. Inspired by the crystalline–amorphous frontier observed in natural biomineralization, Tang et al. designed a novel material based on calcium phosphate ion clusters (CPICs) to induce the epitaxial growth of enamel apatite crystals [164]. They utilized calcium phosphate ion clusters stabilized via triethylamine (TEA) in ethanol to form ultrasmall clusters [165]. These clusters remained stable for a significant period without aggregation or changes in size, providing a promising approach to enamel repair.

**Figure 6:**
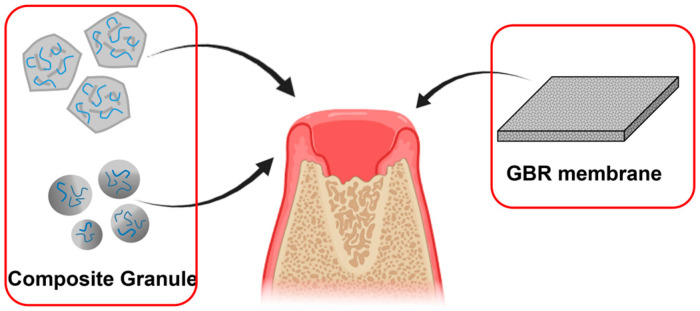
Dental application for alveolar bone regeneration.

Damage to the alveolar bone, such as that caused due to gum disease, can lead to tooth loss and the necessity of dental implants [166]. When there is insufficient alveolar bone, placing implants becomes challenging, requiring the use of grafting techniques to build up the bone (Figure 6). At present, composite grafts made of autologous bone minerals [167], allogenic bone minerals [168], animal-derived bone minerals [169,170], and synthetic bone minerals [171,172] are the most effective methods for regenerating alveolar bone. For instance, researchers devised an enhanced bone regeneration system employing an inorganic–polymer composite material based on biominerals [173]. Traditional alveolar bone regeneration materials, such as HAP, are characterized by low biodegradability and poor drug-loading capabilities [174]. However, by immobilizing silica-forming peptides onto the HAP or β-TCP surface in the presence of silicic acid (Si(OH)_4_), a biosilica-coated ceramic graft is obtained. This facilitates collagen integration onto its surface, resulting in significant improvements in cell adhesion, drug-loading efficiency, and bone regeneration [158,159].

These grafts aid in normal alveolar bone regeneration by occupying the void and precluding soft tissue intrusion [175,176]. In dental surgery, composites are frequently employed to obstruct bacterial or fibroblast invasion (Figure 6). Owing to their porous structure, guided bone regeneration (GBR) membranes in dentistry deter bacteria and fibroblasts from infiltrating into the bone while permitting the passage of nutrients and blood, thus sustaining normal bodily functions [177]. Silica-based silver nanoparticles demonstrated potent antibacterial action against *Streptococcus mutans* without compromising host cell viability, indicating potential for the prevention of dental caries [178]. This mineral composite can be employed in GBR membranes to foster alveolar bone regeneration. Nevertheless, not all polymers are suitable for membrane fabrication. For instance, PLA is extensively used as a material for GBR membranes and scaffolds in periodontal implants [179], where its degradation products exhibit acidic properties with sterilizing effects [180]. However, the alkaline conditions engendered by biomineral admixture can neutralize the acidic milieu, thus establishing a neutral environment conducive to bacterial proliferation on the periodontal membrane [181].

Bleeding, mobility, and peri-implant infections are common challenges associated with dental implants, which can impact the success of the implantation process [182]. Consequently, implant materials must possess biocompatibility and mechanical strength. Anti-inflammatory and antimicrobial properties are also desirable characteristics [182]. Bio-mineral-based composites are inherently biocompatible [183], reducing the risk of adverse reactions and promoting healing and integration with the surrounding tissues [184]. The current research on dental implant design and material selection emphasizes the utilization of biomineralization processes to create biomimetic and biocompatible materials resembling natural hard tissues like bones and teeth [115,185,186]. Although titanium and titanium-based alloys are the dominant dental materials used in dental implants, new materials with altered compositions are being developed to improve existing discomfort; for instance, biological inertness and the surface modification of Ti using BG can improve osteointegration and osteogenesis [187]. Polyetheretherketone (PEEK) is an engineering plastic that offers excellent strength, high biocompatibility, and good chemical stability in most environments [188]. However, the application of PEEK is limited due to its biological inertness, hydrophobicity, and susceptibility to microbial infections [189]. To address these issues, the surface of PEEK can be modified, or additional materials can be incorporated. NaOH-etched/boron-doped nanohydroxyapatite-coated PEEK implants demonstrated an enhanced proliferation and differentiation of osteogenic cells [190]. Copper oxide/silver nanoparticle-decorated PEEK implants show antimicrobial activity and enhanced osteogenesis [191,192].

### 4.3. Artificial Ligament/Tendon Application

**Figure 7:**
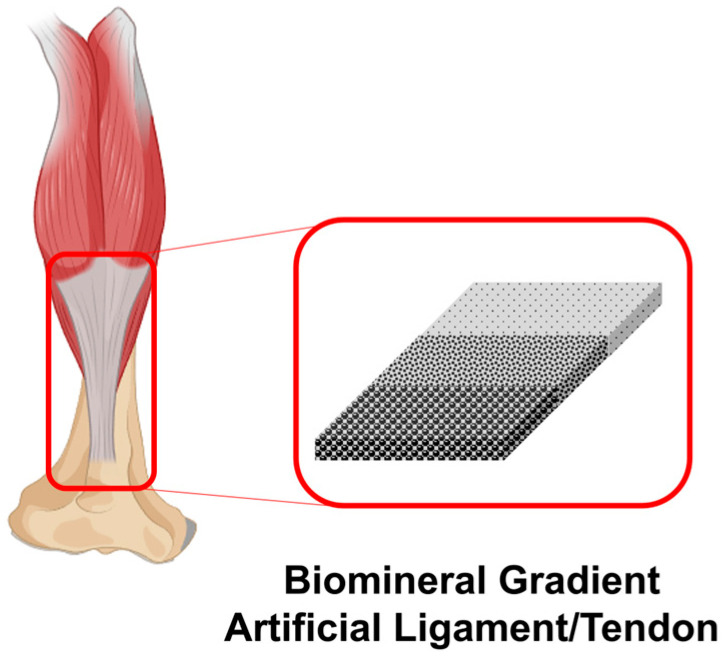
Artificial ligament/tendon using biomineral gradient scaffolds.

Ligaments and tendons are fibrous structures that play a pivotal role in human movement and connecting bone to cartilage [193]. Injuries such as ligament or tendon tears, particularly prevalent among physically active individuals, are characterized by their inability to self-heal. The recovery period for ligament or tendon injuries is significantly longer than that for other musculoskeletal tissues, and the comprehensive rehabilitation of these tissues often exceeds one year [194]. The inherent challenge in rehabilitating ligaments and tendons, which are tissues intimately connected to bones, is compounded when implants are solely constituted of polymers like alginate, collagen, and gelatin [195]. Consequently, repaired ligaments and tendons are invariably weaker than their healthy counterparts, potentially due to the absence of mechanical stimulation during the repair process. Additionally, it is challenging to replicate the complex shapes of tendons and ligaments with polymer materials alone. Finally, polymers do not integrate well with surrounding tissues, which can lead to complications during the healing process. These issues highlight the need for a multi-material approach to effectively repair or replace damaged ligaments and tendons. In comparison to the inferior properties of natural polymers, polymers including ultra-high-molecular-weight polyethylene-reinforced ethylene butene copolymer and polyethylene terephthalate-reinforced polyhydroxyethyl methacrylate facilitate the recreation of the intricate shapes of tendons and ligaments [195,196]. Furthermore, research is being directed towards artificial ligaments and tendons synthesized from biominerals utilizing bone-analogous components. To emulate the connective structures of skeletal muscle and bone, a gradient structure for biomineral particles is employed in artificial ligaments and tendons [197]. Investigations reveal that artificial ligaments/tendon incorporating a gradient structure of HAP exhibit enhanced bone integration in regions with a high concentration, thereby facilitating the recovery of damaged ligaments (Figure 7). Conversely, skeletal muscle attachment is favored in areas with a reduced HAP gradient [198]. Accordingly, one effective strategy has been to augment the surface of artificial ligaments and tendons with bioactive substances or biominerals, thereby enhancing biocompatibility, osteoinduction, and tissue integration. HAP/polymer composites have been successfully developed and evaluated using biomineralization techniques for the construction of artificial ligaments and tendons [199,200]. Nanofibrous scaffolds with a gradient mineral coating were fabricated using 10 × simulated body fluid (SBF) and silk fibroin (SF) [201]. The fabricated scaffolds demonstrated the formation of fibrocartilage-like tissue, indicating that they could enhance the integration at the interface between tendon and bone. magnesium-containing mineral whitlockite (Ca_18_Mg_2_(HPO_4_)_2_(PO_4_)_12_) is a type of calcium orthophosphate crystal in which, under biological conditions, magnesium is partly substituted for calcium [202]. Yuan et al. developed citrate-based, mussel-inspired magnesium whitlockite composite adhesives for bone-to-tendon healing [203]. The composite demonstrated a favorable hemostatic ability, osteoconductivity, and osteo-inductivity, which collectively promoted an environment conducive to bone–tendon healing.

Despite the advances in using biomineral particles for ligament repair, there are still challenges to overcome. For instance, the structures and functions of the native tendon and ligaments are too sophisticated to be directly realized via artificial synthesis [204]. Research is ongoing to further understand the role of biomineral particles in ligament repair and develop more effective strategies for their use.

### 4.4. Wound-Healing Application

Human skin, the largest organ of the body, accounts for approximately 15% of an individual’s body weight and serves as a comprehensive defense mechanism [205]. Beyond its protective role, skin is instrumental in regulating temperature, performing sensory and immune functions, and maintaining fluid balance. The wound-healing process encompasses several stages: coagulation and hemostasis, inflammation, proliferation, and remodeling through wound tissue formation [206]. Skin damage may result from thermal/physical trauma or chronic diseases, leading to wounds, disruptions, or deformities. Untreated epidermal wounds may persist in bleeding, succumb to infection due to contamination, and induce the necrosis of adjacent tissues [207,208]. It is crucial to develop biomaterials that actively support the body’s natural healing process, serving as a protective barrier while being biocompatible with the body’s tissue and promoting overall wound healing [209,210]. Moreover, an ideal wound dressing should absorb exudate and promote hemostasis while maintaining a moist environment and possessing antibacterial properties to aid in wound healing [211].

**Figure 8:**
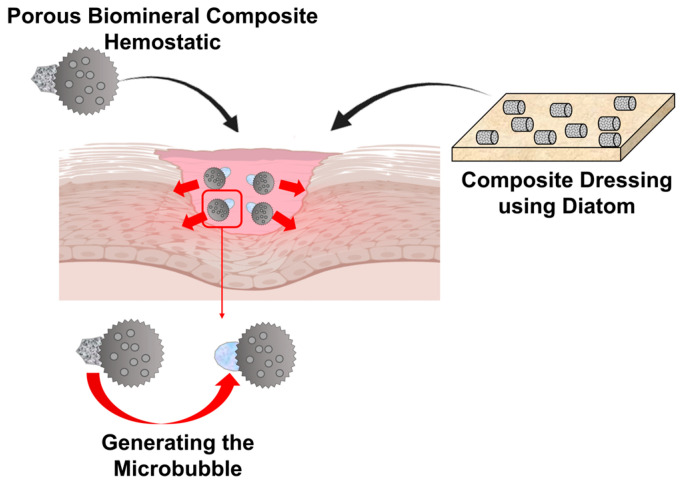
Biomineral-based composite dressing and hemostatic materials for wound healing.

Wound care and management strategies employ an array of polymers, including fibrous proteins and polysaccharides [212]. Biocompatible and biodegradable polymer matrices have been devised to emulate the extracellular matrix, thereby expediting the typically gradual wound-healing process [213]. Such matrices facilitate enhanced cell adhesion, proliferation, migration, and differentiation. Despite the aforementioned advantages, polymer matrices for wound healing can also exhibit certain disadvantages. These include a lack of antimicrobial properties, a deficiency in hemostasis, and a lack of mechanical properties [214]. To augment a dressing’s coagulation, hemostasis, and antibacterial efficacy, researchers have incorporated biominerals like diatoms and calcium carbonate [215,216,217] (Figure 8). Hemostatic agents require the capacity to absorb liquids efficiently in order to control hemorrhaging effectively. This rapid liquid absorption minimizes blood loss, concentrates platelets and clotting factors, and hastens blood coagulation. The diatom, with its hierarchical pore structure, offers an extensive, specific surface area, facilitating a heightened liquid absorption rate. Comprising biosilica, diatoms exhibit significant porosity, which is beneficial for blood absorption and fostering accelerated fibroblast differentiation [218]. Calcium carbonate, utilized in wound dressings, aids in hemostasis. This function is crucial in treating perforating and irregular bleeding, enabling blood coagulation at the sites of hemorrhage. Contemporary strategies aim to enhance hemostatic materials, providing solutions for perforating or irregular wounds by sealing or plugging the skin’s surface to avert hemorrhaging. Traditional hemostatics have shown limitations in wound hemostasis, owing to challenges in their application to irregular or perforated wounds. However, biomineral-based composite materials present an alternative. Janus particles in motor hemostatics, containing CaCO_3_ capable of generating microbubbles, can penetrate deep bleeding sites in perforations and irregular wounds [219] (Figure 8). The generation of macrobubbles via these hemostats permits deeper penetration into bleeding wounds, effectively reducing bleeding. Hence, hemostats utilizing this mechanism prove more efficacious than conventional alternatives. A further composite bound with calcium carbonate has been developed, demonstrating an injectable antimicrobial hydrogel in which calcium carbonate was used as not only a hemostatic agent but also a foaming agent for self-propulsion. This hydrogel, which was composed of quaternized chitosan and exhibited excellent tissue adhesion, a hemostatic effect, fast gelation performance, and an antibacterial effect, is self-expanding, self-propelled, and bioadhesive, with coagulation activity and rapid gelation [220]. It has the potential to be developed as a hemostatic agent for treating catastrophic massive hemorrhage, abdominal organ bleeding, and bleeding from clotting lesions. Consequently, biomineral-based wound dressings have emerged as multifunctional materials endowed with properties conducive to wound healing. This version is structured for academic clarity, detailing the significance of biominerals in enhancing wound healing processes through improved material design and application.

### 4.5. Drug Delivery Application

The process of biomineralization, commonly observed in organisms, involves the merging of inorganic ions with biopolymers to form solid biomaterials. The preparation of nanoparticles, which mimics biomineralization mechanisms, has attracted considerable attention as a potential drug delivery system (DDS) for cancer therapy and regenerative medicine [78]. This is attributed to its uncomplicated and eco-friendly preparation, outstanding biocompatibility, and physical and chemical safety [25]. Controlled DDSs are designed to sustain an effective therapeutic concentration at the targeted site, often within the bloodstream, by facilitating the continuous release of therapeutic agents [221]. This approach enhances patient recovery and comfort while minimizing adverse side effects and toxicity risks.

**Figure 9:**
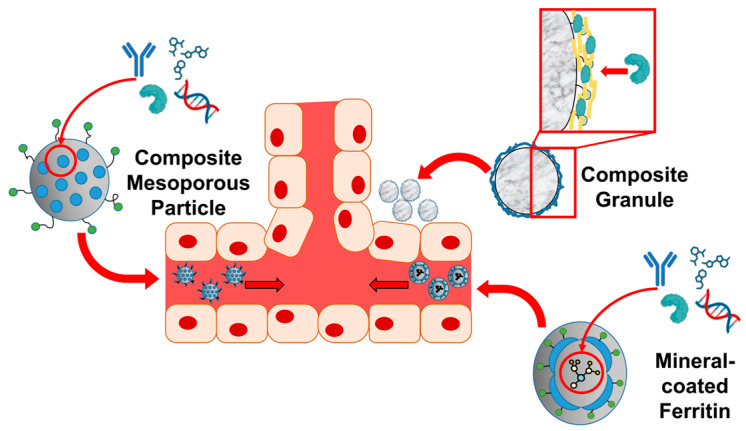
Biomineral-based mesoporous/mineral-coated ferritin/granule composite particles for DDSs.

Traditional silica-based carriers, such as mesoporous silica drug delivery systems (MS-DDS), are well known for their sustained release, nanoscale porous structure, and good solubility for poorly soluble drugs (Figure 9). However, synthesized MS-DDSs can pose challenges such as toxicity in the body, long-term accumulation, and poor excretion due to their acid-driven manufacturing methods [222]. In contrast, biosilica, obtained from diatoms, has recently emerged as an alternative to synthesized MS-DDS. Diatom biosilica has been used for the delivery of anticancer [223,224,225] and antibiotic drugs [226,227]. The structural features of diatoms and the possibility of chemically modifying the frustule allow the transformation of biosilica into potential devices for biomedical applications [228]. Li et al. introduced biohybrid magnetic microrobots based on *Thalassiosira weissflogii* frustules for targeted drug delivery [228]. The biohybrid microrobots have a high drug-loading capacity and demonstrate a pH-sensitive drug release, enhancing their potential for targeted anticancer therapy. Moreover, this microrobot exhibits flexible controllability and environmental adaptability, showcasing various motion modes under a rotating magnetic field, enabling them to navigate through narrow passages and move as a swarm, allowing for precise navigation to specific areas for drug release.

The understanding of biosilica mineralization has led to the discovery and utilization of molecules that can replicate similar functions in the synthesis of biosilica. These molecules are available for fabricating biosilica composite materials. The conventional application of bone morphogenetic protein 2 (BMP2) in conjunction with bone grafts relies on a dipping method through which BMP2 is incorporated into the bone graft prior to clinical usage [159]. The transient nature of BMP2′s binding to bone grafts, primarily through passive adsorption—a form of physical adsorption—results in a substantial initial burst release due to the absence of a binding affinity between BMP2 and the bone graft. Given the high cost of growth factors like BMP2 and their potential side effects upon rapid release into the bloodstream, there is an urgent need for a delivery system that allows for the sustained release of it. BMP2 has been proven to be effective in repairing bone defects when immobilized on a bone scaffold with a biosilica surface to induce the sustained release of BMP2 [158,159] (Figure 9). The silica surface of the bone graft was prepared by first attaching a HAP affinity tag to a silica-forming peptide (SFP), to immobilize it on the HAP surface. The HAP surface was then coated with silica mineral through a peptide-induced silica deposition reaction. This biosilica, which is rich in silanol groups, enhances the binding of growth factors with positively charged heparin-binding motifs compared to HAP [159]. In addition, the BMP2 protein itself has biosilica-forming properties, leading to the development of a BMP2 delivery system through self-encapsulation via BMP2-mediated silica synthesis [75,76]. BMP2/silica composites have demonstrated the sustained release of BMP2 from bone grafts.

Pack et al. introduced a biocompatible DDS that amalgamates protein nanocages such as ferritin (Fn) with bio-inspired silicification (Figure 9). Fn is a large, spherical protein composed of 24 monomers, measuring 12 nm in total size with an internal space of 8 nm. It has the capacity to hold up to 4500 iron atoms, converting harmful iron into a safe form to protect cells from damage [229]. Once the iron ions are removed, Apo-Fn can serve as a cage for drug delivery. Furthermore, the outer surface can be modified to attach cancer-targeting ligands or antibodies, while fluorophores can also be added for tracking purposes [230]. By incorporating an SFP into the Fn outer surface, the system directs the deposition of biosilica around the Fn-SFP fusion proteins [77,231,232]. These Silica/Fn-SFP nanoparticles exhibited high drug-loading capacities along with pH-responsive drug release patterns and a dual DDS [232]. Leveraging this biocompatible framework, a dual DDS was developed, capable of concurrently administering anticancer drugs and any other type of drug, such as antibiotics, thus effectively combating cancer and microbial infections [35,233,234].

DNA nanoframeworks possess great biological information and controlled framework structures, making them highly promising for various biological applications [235,236]. However, unmodified DNA nanoframeworks are susceptible to various forms of degradation, including hydrolysis, depurination, depyrimidination, oxidation, alkylation, or nuclease degradations, limiting their stability and applicability [237]. To address this issue, Wang et al. have successfully stabilized DNA nanoframeworks through biomineralization with silica, resulting in silica-armored DNA nanoframeworks (Si-DNA nanoframeworks). This enhancement has rendered them suitable for efficient intracellular delivery, and it has effectively prevented degradations and leakages of loaded reagents such as siRNA and doxorubicin [237].

Porous calcium carbonates and phosphates have demonstrated their potential as effective drug carriers due to their biocompatibility and biodegradability [238,239,240]. Miyamaru et al. designed and synthesized peptide lipids with specific sequences, which were then incorporated into vesicles and mineralized to create CaCO_3_-coated vesicles [241]. The mineralized vesicles retained their spherical shape and had inner spaces for drug cargo, with the CaCO_3_ shell dissolved under weakly acidic conditions, such as a pH of 6.0 to release the encapsulated drugs, making them potential candidates for cancer therapies. Huang et al. demonstrated a one-pot, L-lysine (Lys)-mediated biomineralization method using a CO_2_ bubbling procedure to prepare CaCO_3_-based DDS [242]. The presence of Lys enhanced the yield of CaCO_3_ and controlled the morphology and crystal phase of the CaCO_3_. A further study on the control of the calcium carbonate structure using amino acids demonstrated the efficacy of sound waves (ultrasonic irradiation) and L- or D-aspartic acid in the creation of small, uniquely shaped calcium carbonate particles, which are more suitable for medical applications such as drug delivery and bone healing [243]. Gene therapy is an important treatment for complex diseases, and inorganic CaCO3 nanoparticles have been utilized for efficient gene delivery. By varying the ratio of Ca/CO_3_^2−^, these nanoparticles can efficiently load DNA and drugs with high encapsulation efficiency, enhancing their potential for gene therapy [244].

A novel strategy to block cancerous tissue through biomineralization has been reported [245]. The research introduced a biomineralization-inducing nanoparticle (BINP) for blockade therapy to treat osteosarcoma. BINP is composed of dodecylamine-poly((γ-dodecyl-l-glutamate)-co-(l-histidine))-block-poly(l-glutamate-graft-alendronate), and it combines a cytomembrane-insertion moiety, a tumor-micro-environment (TME)-responsive component, and an ion-chelating motif. The acidic environment of a tumor triggers the exposure of the dodecyl group on the surface of the expanded BINP, facilitating their cytomembrane insertion. Subsequently, the protruding bisphosphonic acid group on the BINP triggers continuous ion deposition with calcium ions derived from TME to construct a mineralized barrier around the tumor. This mineralized barrier blocks the substance exchange between the tumor and the surrounding normal tissues, contributing to the efficacy of blockade therapy for osteosarcoma. The selective biomineralization initiated via BINP provides a promising alternative for clinical osteosarcoma therapy and the further inhibition of pulmonary metastases.

A summary of some applications of biomineral-based composites in regenerative medicine is shown in Table 1.

## 5. Challenges and Future Directions

### 5.1. Current Challenges in the Use of Biomineral Composite Materials in Regenerative Medicine

Biomineral-based composites have emerged as a promising class of materials in the field of regenerative medicine. These materials, which combine organic and inorganic components, mimic the natural mineralized tissues found in the human body and nature. Despite their potential, the use of biomineral-based composites in regenerative medicine is not without its challenges.

One of the primary challenges in the use of biomineral-based composites is achieving the desired mechanical properties. The mechanical strength and durability of these composites are crucial for their performance in regenerative medicine applications [36]. However, achieving the right balance between strength and flexibility can be challenging. The mechanical properties of biomineral-based composites such as stiffness, pore sizes, surface topology, and load-bearing capacity [246] can be influenced due to several factors, including the choice of materials, the fabrication process, and the microstructure of the composite [36]. Biocompatibility is another critical factor in the use of biomineral-based composites in regenerative medicine. The materials used in these composites must be compatible with the human body and should not elicit any adverse immune response. While many biomineral-based composites exhibit good biocompatibility, others may cause inflammation or other immune reactions [247]. Biodegradability is also a significant consideration. Ideally, a biomineral-based composite used in regenerative medicine should degrade at a rate that matches the rate of tissue regeneration. Biodegradability allows the release of ions or drugs such as growth factors and antibiotics that could help promote bone tissue regeneration as well [159,241,248,249]. However, controlling the degradation rate of these composites can be challenging. Reproducing the hierarchical structures found in natural tissues is another challenge in the use of biomineral-based composites in regenerative medicine. Highly interconnected porous structures that allow vascularization, cell migration, delivery of nutrients, and tissue ingrowth are required for successful tissue regeneration [250]. While advances in fabrication techniques, such as 3D printing, have made it possible to create complex structures, there is still much work to be done in this area. The mass production of biomineral-based composites for regenerative medicine applications presents its own set of challenges. Scaling up the production process while maintaining the quality and performance of the composites can be difficult. Li et al. presented a mild approach to producing large-scale, structurally colored composite films by shearing supramolecular composites composed of polymers and colloids with supramolecular interactions [251]. The study elucidates the mechanism of colloidal ordering during the shearing of supramolecular composites, providing valuable insights into the process of producing ordered composites within minutes, which is beneficial for the scale-up production of mineral colloid and polymer composites. The ease of fabrication is another important consideration in the use of biomineral-based composites [252]. The fabrication process should be straightforward and reproducible. However, this is not always the case, and the fabrication of biomineral composites can be a complex and time-consuming process. Additionally, the environmental impact of biomineral-based composites is a growing concern. These materials should be environmentally friendly in terms of both their production and their disposal. However, achieving this goal can be challenging, given the complex nature of these composites. Finally, to make it practical, we must address challenges such as the need for standardized material processing, ensuring long-term safety, navigating strict regulatory pathways, and reducing manufacturing costs [253]. The regulatory landscape for these materials is complex and can pose significant hurdles to their widespread adoption. Basically, in order to conduct clinical trials and receive approval as a medical product, they must provide a certain form or effect and provide biological stability. However, in the case of composite materials that combine multiple materials and drugs, it is difficult to verify leachability, extracts, and effects. Additionally, it is difficult to predict side effects in areas other than the target tissue [254]. Overcoming these hurdles requires collaborative efforts across different disciplines and strategic regulatory planning.

### 5.2. Future Research Directions

This section presents an overview of potential avenues for future research in the field of biomineral-based composites. It focuses on the development of the design of innovative composites, and the advanced composite manufacturing technologies.

Bio-inspired mineralization methods have become a promising approach to creating biomineral-based composites used in regenerative medicine [36,255]. These methods, inspired by nature’s remarkable ability to form intricate minerals, offer numerous advantages over traditional methods, including easy mineral deposition, environmentally friendly synthetic processes, and no or fewer by-products [36,255]. One of the key advantages of bio-inspired mineralization methods is the ease of mineral deposition. By mimicking how organisms orchestrate mineral growth, researchers have been able to create next-generation materials with better mechanical properties and stability through ingenious and reasonable synthetic strategies [255,256]. Bio-inspired mineralization is a low-energy and environmentally friendly process, as it synthesizes minerals at room temperature, normal pressure, and a neutral pH, in contrast to chemical synthesis, which requires harsh conditions such as high temperatures and extreme pH [257]. Bio-inspired mineralization methods also minimize the production of by-products. This approach not only reduces waste but also makes the production process more sustainable. Biocompatibility is another significant advantage of bio-inspired mineralization methods. Many biomineral composites exhibit good biocompatibility, making them suitable for diverse biomedical applications [258,259]. Biominerals produced through bio-inspired mineralization are organic/inorganic composites that closely resemble natural biominerals, rendering them biodegradable [260].

Biominerals display complex hierarchical architectures, such as nacre, bones, and frustules in diatoms, which are difficult to replicate using synthetic materials due to manufacturing constraints and the extensive range of potential configurations in the design space [36,257]. Artificial intelligence (AI) and machine learning (ML) techniques have the potential to greatly optimize the manufacturing process of biomineral-based composites. The ML model is trained with a database of hundreds of thousands of structures from finite element analysis, together with a self-learning algorithm for discovering high-performing materials. The results show that this approach can create microstructural patterns that lead to tougher and stronger materials [261]. Scientists have examined complex formations with customized characteristics and adaptable reactions by utilizing additive manufacturing (AM) techniques, such as AI-guided 3D printing and 4D printing [262]. This approach expedites simulations, optimizes material selection, facilitates the design of new structures with multiple functionalities, and diminishes both time and costs. Yu et al. presented a technique for creating materials with complex variations in their microstructure, taking inspiration from the development of natural materials to achieve enhanced mechanical properties [263]. They developed a new method that employs reinforcement learning to autonomously arrange microstructures using both brittle and soft materials. This method adopts a hierarchical, multiresolution design approach, gradually enhancing the resolution of the design space to achieve higher resolutions. Nacre exhibits hierarchical structures that offer an exceptional balance of multiple material properties, making them an ideal candidate for biomimetic design. Park et al. presented a data-driven framework that utilizes Gaussian process regression and multi-objective Bayesian optimization to create bio-inspired composites with an ideal combination of material properties [264]. Their approach was specifically applied to a nacre-inspired composite, resulting in the generation of a 3D Pareto surface for optimal design. This design was subsequently validated through the tensile testing of 3D-printed samples. A mixed method that combines machine learning algorithms, virtual sample generation, physics-based simulations, and experimental data can be used to improve automated fiber placement (AFP) in the production of thermoplastic composites [265]. Chiu et al. utilized a genetic algorithm (GA) and conditionally variable automatic encoder (CVAEs) to analyze a binary composite system with edge cracks and a lattice-like structure [266]. Their objective was to develop composites that possess specific stiffness and toughness properties. They discovered that, through the placement of a soft material behind the cracks, the material’s stiffness was significantly increased. They also conducted real-world tests on 3D-printed versions to validate their computer predictions. Kim et al. introduced a forward-design approach based on deep neural networks to effectively search for superior materials outside the initial training set [267]. This method enables the efficient exploration of new materials within a wide design space. Their framework demonstrates the ability to generate high-quality designs approaching the global optimum, even with the addition of very small datasets comprising less than 0.5% of the original training dataset size. These studies demonstrate the capacity of AI and machine learning to enhance the development and production of biomineral-based composites. These advancements provide insight into the potential of AI and ML in the field of materials science, where they could be instrumental in the exploration and creation of new materials with enhanced properties. Nevertheless, it is crucial to acknowledge that this is a swiftly progressing domain, and further improvement, including the availability of high-quality, labeled data, advanced machine learning algorithms, data augmentation, and integration with domain knowledge [262,268], is required to completely comprehend the capabilities of these technologies.

Biomineralized materials are complex material systems that have intricate, three-dimensional material structures. Conventional techniques lack the ability to reproduce the hierarchical organization and flexibility found in natural materials [269,270]. Three-dimensional printing techniques are employed in the manufacture of advanced, multifunctional polymer composites due to their mass customization, freedom of design, capability to print complex, 3D structures, and rapid prototyping [271]. However, the production of extensive, accurate ceramics that can efficiently regulate the bioactivity of the material continues to be a difficult task. Zhang et al. utilized Digital Light Processing (DLP) to manufacture hydroxyapatite porous bioceramics on a large scale, with a length exceeding 150 mm [272]. The resulting bioceramics exhibited a highly intricate, porous surface structure with a printing resolution of less than 65 μm. The results indicate that DLP technology has the capacity to create bone tissue engineering scaffolds on a large scale while maintaining precise porosity [272]. Three-dimensional printing technology significantly impacts the biomineral composition of composites by enabling the fabrication of structurally complex materials with enhanced properties. Through the integration of 3D printing with biomineralization processes, such as enzyme-induced mineralization, hydrogel architectures can be transformed into rigid and highly mineralized composites, allowing for precise control over mineral distribution within constructs [269]. This advancement facilitates accelerated and homogeneous biomineralization, leading to the deposition of minerals on scaffold surfaces and promoting bone regeneration at defect sites [273]. The combination of 3D bioprinting and nano-biomaterials in tissue engineering and regenerative medicine has resulted in the creation of intricate cellular structures through bio-fabrication [274]. Nanometer-scale bioactive material possesses the ability to guide the development of cells, influencing their specialization and contributing to the creation of functional structures [273,274]. Three-dimensional printing has revolutionized the processing of biomaterials by making it feasible to construct intricate structures with high resolution and precision. However, achieving the desired precision and resolution can be challenging, especially when dealing with complex structures and materials [275]. For example, metal or ceramic additive manufacturing methods have been used to reproduce the tough component of biomineral-based composites. Nevertheless, imperfections that occur during the printing or post-processing stages, such as porosity, inhomogeneities, and sintering defects, generally lead to printed products that are less strong than their solid counterparts. Moreover, numerous manufacturing processes require conditions of elevated temperature and/or elevated pressure. These challenges offer prospects for additional investigations and advancements in the domain of 3D printing for biominerals. Successfully addressing these obstacles has the potential to result in notable progress in the field of biomedical applications.

## 6. Conclusions and Perspective

Biominerals are composite materials created by living organisms through biomineralization. They are known for their biocompatibility, self-healing, flexibility, hierarchical architectures, light weight with high mechanical strength, and elasticity. Biomaterials for hard tissue regeneration are often derived from biominerals, but issues such as mass production, immune rejection, and viral contamination have prompted research into biomineral-based composite materials produced through mimicking biomineralization. Biomineral-based materials, including calcium- and silica-based minerals, have had a significant impact on regenerative medicine due to their biocompatibility and bioactivity. They facilitate key cellular functions such as adhesion, proliferation, and differentiation. Biominerals, with their structural specificity, provide an extensive surface area and enable the addition of extra functionalities via surface functional groups. By incorporating surface functions, drugs can be transported more effectively, resulting in a reduction in side effects and costs. This is achieved by suppressing the burst release. Moreover, it has been designed to carry out a physical function called a motor, which makes it highly effective in treating wounds. The application of composite materials can overcome the limitations associated with traditional regenerative materials that rely on a single component. Biodegradable biominerals used within the human body can continuously release essential ingredients for tissue regeneration, and they have been confirmed to enhance the regeneration of hard and soft tissues by mimicking the micro-environment.

Challenges in the field of biomineral-based regenerative medicine include issues such as replicating nature’s hierarchical architectures and maintaining mechanical properties during the regeneration process while ensuring biodegradability for the integration of a newly regenerated tissue with an existing one. Furthermore, the development of low-cost, simple, and environmentally friendly fabrication processes for biomineralized composites is essential for the mass production and clinical dissemination of these materials. In addition, challenges such as standardized material processing, long-term safety assurance, navigating stringent regulatory pathways, and reducing manufacturing costs need to be addressed. Overcoming these hurdles will require collaborative efforts across different disciplines and strategic regulatory planning.

Looking ahead, the outlook for biomineral-based regenerative medicine is promising. Nanotechnology, 3D-printing technology, and smart biomaterials hold the potential to further enhance the functionalities of these materials, leading to programmable properties and improved tissue integration. Efforts are being made to develop biomineral materials that mimic natural biominerals to enhance therapeutic effects, and there is potential for the use of innovative technologies beyond bone and tooth repair. Collaborative efforts across disciplines are essential to overcoming the current challenges and expanding the use of biomineral-based treatments to a wider range of medical fields. The integration of biominerals into composite materials offers a way to overcome the limitations of traditional regenerative materials, leading to more effective treatments for tissue damage and pharmacological needs. Ongoing research is refining biomineral-based composites and their roles in tissue regeneration. Future breakthroughs in biomimetics, drug delivery, and tissue regeneration are anticipated, with the potential to transform patient care and enhance one’s quality of life.

## Figures and Tables

**Table 1:** A summary of some applications of biomineral-based composites in regenerative medicine.

	Base Material		Biomineral for Composite	Structural Composite/ Fabrication	Potential Advantages	Ref.
Bone	Non-corrosion metal	Electrospun PCL nanofibers@titanium	Carbonated HAP nanoparticles	Coating	Improve cell adhesion, corrosion resistance, and overall implant properties	[143]
	Synthetic polymer	PCL	BG	Doping	High cell-response values and toughness	[149]
		Polyphosphazene	HAP	Solvent casting, melt blending, or in situ polymerization	Enhanced bioactivity, biocompatibility, and osteoconductivity	[155]
		PLLA	HAP	HAP absorbed on porous, 3D-printed PLLA screw	Increase the inductivity of bone, promote bone growth in the bone tunnel, and promote bone integration at the tendon–bone interface	[156]
	Natural polymer	Gelatin	Biosilica, CaCO_3_	Immobilization on electrospun fiber	Improve cell attachment and bone differentiation	[152]
		Chitosan, collagen, silk fibroin, hyaluronic acid, and gelatin	Nano-HAP	Freeze-drying	Enhanced cellular attachment, survival, and osteogenic differentiation Improved mechanical properties	[154]
		Collagen	Biosilica/β-TCP	Embedded in collagen	Improve bone regeneration	[158]
Dental	Ceramic granule	HAP	Biosilica	Coating	Enhanced BMP2 delivery and bone regeneration	[159]
		HAP TEA and ethanol	Amorphous calcium phosphate Calcium phosphate ion clusters	Epitaxial growth of enamel apatite crystals	Similar morphological texture and mechanical strength between the repaired layer and native enamel	[164]
		BMP2	Autologous bone	Adsorption	Enhanced bone growth	[167]
		Polymer and gelatin	Bovine bone mineral	Xenograft enriched with gelatin and a polymer	Higher proportion of lamellar bone and osteoid	[170]
		Calcium phosphate PCL	Calcium phosphate Mg, Zn, Sr	Spin coating	Faster dissolution rate	[185]
	Nanoparticles	Silver nanoparticles	Silica-coated silver nanoparticles	Coating	Biocompatible and antimicrobial	[178]
	Polymer	Collagen	Magnesium-doped hydroxyapatite	Embedded	Strong cell–material interaction	[114]
		Polyacrylic acid, carboxymethyl chitosan, and dentin matrix	Calcium phosphates	Embedded in hydrogel	Self-repairing ability, injectability, and the promotion of odontogenesis and osteogenic differentiation	[186]
	Implants	Ti implants	BGs	Coating	Excellent cell compatibility, antibacterial and anti-inflammatory properties, and higher levels of osseointegration and osteogenesis	[187]
		PEEK implants	Nano-HAP	Coating	Enhanced proliferation and differentiation of osteogenic cells	[190]
Tendon/ Ligament	Synthetic polymer	PCL/chitosan	Nano-HAP	Embedded via an in situ sol-gel process	Enhancement of morphological, mechanical, and biological properties in favor of tendon and ligament regeneration	[200]
		Citrate-based, mussel-inspired adhesive Prepolymer (PEG-PPG-PEG)	Magnesium whitlockite	Embedded in injectable adhesive	Hemostatic ability, osteoconductivity, and osteo-inductivity Promote a conducive environment for bone-tendon healing	[203]
	Natural polymer	Silk fibroin	SBF	Gradient coating	Bone marrow mesenchymal stem cell growth and differentiation Improved osseointegration	[201]
Wound healing	Natural polymer Synthetic polymer Protein	Hydroxybutyl chitosan	Diatom biosilica loaded with doxycycline	Coating	Improve hemostasis and hemorrhage High loading capacity and sustained release of doxycycline Antimicrobial activity	[215]
		Chitosan	CaCO_3_	Embedded	Instant hemostasis accelerated wound healing	[217]
		Negatively modified, microporous starch	CaCO_3_	Flower-shaped calcium carbonate crystals uniaxially grown on microporous starch	Rapid hemorrhage control of deep bleeding sites	[219]
		Oxidized dextran Quaternized chitosan	CaCO_3_	Embedded in hydrogel in the form of oxidized detran/CaCO_3_ mixture	Hemostatic CO_2_ forming	[220]
Drug delivery	Mineral	BMP2	Biosilica	Coprecipitates	Enhanced BMP2 delivery and osteogenesis	[75,76]
	Diatom	Diatom biosilica	Fe_3_O_4_ magnetic nanoparticle	Attachment to diatom biosilica	Controllable with magnets	[228]
	Cage protein	Ferritin	Silica	Silica coating	Controllable drug delivery	[77,231,232]
	DNA	DNA nanoframework	Silica	Silica coating	Effectively prevent degradations and leakages of loaded siRNA and doxorubicin	[237]
	Vesicles	Vesicles embedded with the peptide lipids	CaCO_3_	CaCO_3_-coated vesicles	pH-controlled release	[241]
	Amino acid	L-Lysine	CaCO3	l-lysine-mediated CaCO_3_ synthesis	Significant differences in drug-loading rate, loading capacity, and pH sensitivity due to differences in crystal form and morphology	[242]
		L-aspartic acid, D-aspartic acid	CaCO_3_	Chiral-curved CaCO_3_	Control of morphology and size of CaCO_3_,	[243]
	Peptide	CPP-KR12	Biosilica	Coprecipitates	Reduced cytotoxicity Enhanced antimicrobial peptide delivery	[35]
		Dodecylamine-poly((γ dodecyl-l- glutamate)- co-(l-histidine))-block-poly(l-glutamate-graft-alendronate)	Calcium phosphate	Coprecipitates via ionic interaction	Blockade therapy for osteosarcoma and inhibition of pulmonary metastases	[245]

## Data Availability

Not applicable.
